# Salidroside exerts anti-tumor effects in ovarian cancer by inhibiting STAT3/c-Myc pathway-mediated glycolysis

**DOI:** 10.17305/bb.2024.10867

**Published:** 2024-08-02

**Authors:** Ge Yu, Xiaoling Feng

**Affiliations:** 1 Gynecology Department, Harbin Medical University Cancer Hospital, Heilongjiang, China; 2 Gynecology Department, First Affiliated Hospital Heilongjiang University of Chinese Medicine, Heilongjiang, China

**Keywords:** Ovarian cancer (OC), salidroside (SAL), signal transducer and activator of transcription 3 (STAT3)/c-Myc pathway, glycolysis

## Abstract

Salidroside (SAL) is a bioactive substance extracted from the traditional Chinese medicine *Rhodiola rosea*, which exhibits multiple pharmacological effects, such as anti-inflammatory, antioxidant, and anti-tumor properties. Currently, the effects of SAL on the malignant progression of ovarian cancer (OC) and its specific mechanism of action are not clear. Cell counting kit 8 (CCK-8), clone formation, Hoechst 33258 staining, flow cytometry, Transwell, western blotting, and immunofluorescence assays were performed to determine the impacts of SAL on the biological properties of OC cells (CAOV3 and SKOV3) and human normal ovarian epithelial cells (IOSE80). The binding activity of SAL and proteins was evaluated. Glucose consumption, lactate and ATP production, extracellular acidification rate (ECAR), and related proteins were measured to assess glycolysis. Animal models were established to evaluate the impact of SAL treatment in vivo and the expression levels of signal transducer and activator of transcription 3 (STAT3)/c-Myc pathway-related proteins were determined to explore the relationship between SAL and OC. The results showed that SAL reduced the viability, clone formation, migration, and invasion ability of CAOV3 and SKOV3 cells, and induced apoptosis. SAL inhibited epithelial–mesenchymal transition (EMT) and decreased glucose consumption, lactate and ATP production, and ECAR. SAL exhibited good binding activity with STAT3 and c-Myc and reduced the expression levels of STAT3/c-Myc pathway and glycolysis-related proteins in vitro and in vivo. In conclusion, SAL exerted anti-tumor effects by interfering with the malignant biological progression of OC cells by inhibiting STAT3/c-Myc pathway-mediated glycolysis.

## Introduction

Ovarian cancer (OC) is a commonly diagnosed malignant tumor of the female reproductive system and is the second most frequently occurring cancer in this system, with uterine cancer being the first [1, 2]. According to statistics, OC accounts for 5% of female cancer deaths, with up to 300,000 new cases and over 150,000 deaths occurring annually [3, 4]. OC has an insidious and aggressive onset, with most patients being diagnosed in the middle to late stages, and more than 50% of patients experience recurrence within two years, leading to a poor prognosis [5–7]. Although radiotherapy, platinum-based chemotherapy, and targeted therapies for OC continue to evolve, a significant portion of patients still have difficulty benefiting from existing treatments due to low response rates, toxic side effects, and resistance to treatment [8, 9]. In addition, immunotherapy, such as adoptive cell transfer and immune checkpoint inhibitors, has gained increasing attention in tumor treatment, but it may come with immune-related toxicities and thyroid dysfunction as side effects [10, 11]. In view of these, the search for novel and efficient drugs is crucial for the clinical management of patients with OC.

Over the past few years, herbal extracts have garnered significant attention for their ability to hinder the malignant advancement of tumors, due to their high safety levels, low side effects, multiple pathways, and multiple targets [12, 13]. *Rhodiola rosea* is a multifunctional plant with edible and medicinal values, mainly found in the Himalayas, northwestern Asia, and North America [14, 15]. Salidroside (SAL) is a phenylpropane glycoside extracted from the *Rhodiola rosea* plant, which exhibits a range of pharmacological effects, such as cardiovascular system protection, reducing inflammation, antioxidant, anti-aging, and anti-tumor properties [16, 17]. In recent years, the anticancer function of SAL has received extensive attention from scholars, and a multitude of studies have confirmed that SAL has the potential to suppress the malignant progression of tumors, such as prostate cancer [18], non-small cell lung cancer [19], gastric cancer [20], and pancreatic cancer [21]. Yu et al. [22] reported that SAL could induce apoptosis in OC cells and may be a promising new anti-OC drug. However, the specific mechanism of action of SAL in inhibiting the malignant progression of OC is not clear.

With its numerous biological functions, the signal transducer and activator of transcription 3 (STAT3) is a key transcription factor, which is closely associated with the apoptotic process of tumor cells and has the ability to increase the resistance of tumor cells to drug toxicity [23, 24]. c-Myc regulates glycolysis in tumor cells, thereby promoting the Warburg effect, and plays a key role in a variety of tumors [25, 26]. Therefore, this research aimed to investigate the influence of SAL on OC cell proliferation, migration, invasion, epithelial–mesenchymal transition (EMT), and glycolysis, and to determine whether SAL regulates OC malignant progression by the STAT3/c-Myc pathway. The goal of this research is to provide a reference for the application of SAL in the clinical treatment of OC.

## Materials and methods

### Cell culture and treatment

Human normal ovarian epithelial cells (IOSE80) were sourced from the Shanghai Cell Bank of the Chinese Academy of Sciences. OC cell lines (CAOV3 and SKOV3) were obtained from Pricella Biotechnology Co., Ltd (Wuhan, Hubei, China). In sterile culture flasks, cells were nurtured using RPMI-1640 medium (Gibco, Grand Island, NY, USA) along with 10% fetal bovine serum (Gibco) and 1% penicillin/streptomycin (Gibco). The culture was maintained at 37 ^∘^C with 5% CO_2_. The cells were subcultured every three days and refreshed with a new culture medium every other day.

In cell viability assay experiments, SAL (43866, Sigma-Aldrich, St. Louis, MO, USA) concentrations were 0, 50, 100, 200, 400, or 800 µM. In subsequent cell biological characterization assays, SAL concentrations were 200, 400, or 800 µM. For the SAL+Colivelin TFA group, cells were co-intervened with 800 µM of SAL and 50 µg/mL of Colivelin TFA (HY-P1061A, MedChemExpress, Monmouth Junction, NJ, USA) for 24 h.

### Cell counting kit-8 (CCK-8) assay

IOSE80, SKOV3, and CAOV3 cells were inoculated in 96-well cell culture plates (4.0×10^4^cells/well). After the cells adhered to the walls, the original medium was discarded and replaced with a medium with varying levels of SAL or SAL+Colivelin TFA. Following the next 24 h, a complete medium (100 µL) with 10% CCK-8 reagent (C0038, Beyotime, Shanghai, China) was dispensed into each well, and then cultured in the dark (37 ^∘^C, 3 h). The OD_450_ value of the cells was evaluated utilizing a microplate reader (Thermo Fisher Scientific, Waltham, MA, USA).

### Clone formation assay

CAOV3 and SKOV3 cells were digested with trypsin, resuspended, and counted. Then, 500 cells were placed in each well of a 6-well cell culture plate, respectively, and incubated for 14 days (37 ^∘^C, 5% CO_2_). The culture was terminated when the clonal cell mass was visible to the naked eye and rinsed twice with PBS. 4% paraformaldehyde (Solarbio, Beijing, China) was used for fixation, and this process was carried out for 20 min. After discarding the fixative, the cells were treated with 0.1% crystal violet (Sigma-Aldrich) and photographed for counting.

### Hoechst 33258 staining

CAOV3 and SKOV3 cells were inoculated in a 12-well cell culture plate and incubated for 24 h. Then, the cells were treated with 4% paraformaldehyde for 30 min and rinsed three times with PBS. Hoechst 33258 staining solution (C1017, Beyotime) was added and incubated for 10 min, and then the cells were examined for their apoptotic status using a fluorescence microscope.

### Flow cytometry

CAOV3 and SKOV3 cells were taken and centrifuged at 3000 *g* for 8 min, then rinsed three times with PBS and resuspended in Binding Buffer (500 µL). Annexin-V-FITC (5 µL, MedChemExpress) and propidium iodide (5 µL, Beyotime) were added and mixed gently, strictly avoiding the light, and incubated at room temperature for 15 min. Flow-specific sampling tubes were used to transfer the samples, and apoptosis was then identified through flow cytometry.

### Transwell assay

Matrigel dilution gel (100 µL, Sigma-Aldrich) was diluted with serum-free RPIM-1640 medium, then 100 µL of the gel was added to each Transwell (Corning, Tewksbury, MA, USA) and placed in the incubator overnight. The next day, the remaining liquid in the chambers was removed and replaced with serum-free RPMI-1640 medium to rehydrate the basement membrane. CAOV3 and SKOV3 cell suspension (200 µL) was put in the upper chamber, followed by the addition of the right amount of RPMI 1640 medium in the lower section, and then incubated for a period of 36 h. The upper chamber was emptied of the Matrigel gel and cells, and 4% paraformaldehyde (Solarbio) was used for fixation, then treated with 0.1% crystal violet aqueous solution. The number of invasive cells was ascertained.

The method used for the migration experiment closely resembled that of the invasion experiment, except that Matrigel was not added to the upper chamber.

### Western blotting

Protein extraction from cells or tissues was performed by lysing with RIPA lysate (Beyotime), then utilizing the BCA kit (P0012, Beyotime) to determine protein concentrations. The supernatant was taken as protein samples after denaturation. The samples were transferred to PVDF membranes (Invitrogen) and blocked for 2 h following the gel electrophoresis procedure. After rinsing the membrane, it was placed at 4 ^∘^C for an overnight incubation with E-cadherin primary antibody (ab231303, 1:1000, Abcam, Cambridge, MA, USA), Vimentin primary antibody (ab24525, 1:10000, Abcam), Snail primary antibody (MA5-14801, 1:1000, Invitrogen), glucose transporter type 1 (GLUT1) primary antibody (MA5-31960, 1:5000, Invitrogen), hexokinase 2 (HK2) primary antibody (ab227198, 1:5000, Abcam), lactate dehydrogenase A (LDHA) primary antibody (PA5-27406, 1:500, Invitrogen), p-STAT3 primary antibody (710093, 1:100, Invitrogen), STAT3 primary antibody (MA1-13042, 1:5000, Invitrogen), or c-Myc primary antibody (13–2500, 1:200, Invitrogen). The following day, after being rinsed three times, the membrane was cultured with goat anti-rabbit secondary antibody IgG (31460, 1:10,000, Invitrogen), and exposed after development. Image J software was utilized to obtain the grayscale value of each protein band, with β-actin (MA1-140, 1:5000, Invitrogen) serving as the internal reference.

### Immunofluorescence

CAOV3 and SKOV3 cells were grown on laser confocal petri dishes until they reached 50%–60% confluency, after which they were rinsed three times with PBS and treated with 4% paraformaldehyde for 15 min. The cells were covered with drops of 0.3% Tritonx-100 (Sigma-Aldrich) and permeabilized for 10 min. After that, cells were sealed for 30 min with 5% bovine serum albumin (Sigma-Aldrich). The Snail primary antibody (MA5-14801, 1:250, Invitrogen) was left to incubate overnight at 4 ^∘^C. The following day, the secondary antibody sheep anti-rabbit IgG (A-11011, 1:500, Invitrogen) was added and left to incubate for 1 h at 37 ^∘^C in the dark. Finally, after adding the DAPI staining solution (Solarbio), the mixture was incubated in a dark room for 10 min, and the results were observed by laser confocal microscopy within 1 h.

### Glucose consumption, lactate and ATP production measurements

CAOV3 and SKOV3 cells were cultured in a 6-well plate (1.0×10^6^cells/well). The glucose concentration of the culture medium was detected by using a glucose assay kit (S0201S, Beyotime) to determine the glucose consumption by comparing it to the control group. The lactate content assay kit (A019-2-1, Nanjing Jiancheng Bioengineering Institute, Nanjing, Jiangsu, China) and ATP assay kit (S0027, Beyotime) were used to measure lactate and ATP production in the culture medium.

### Extracellular acidification rate (ECAR) detection

CAOV3 and SKOV3 cells were cultured in a 6-well plate (1.0×10^6^cells/well). The complete medium was removed from the well plates, and the well plates were washed three times with base medium (Agilent Technologies, Santa Clara, CA, USA), and then 500 µL of base medium was added to each well. The experimental procedures were performed according to the instructions of the XFe Seahorse glycolysis stress test kit (103020-100, Agilent Technologies), and the ECAR of the cells was measured using the XFe Seahorse energy analyzer (Agilent Technologies).

### Subcutaneous tumors in nude mice

The BALB/c nude female mice used in this study were obtained from Vitalriver (Beijing, China) and kept in a constant temperature environment at 22 ^∘^C and humidity of 55%–60%. Each mouse was subcutaneously injected with 200 µL of logarithmic growth phase SKOV3 cell suspension (2×10^6^ cells/mouse). After seven days, the nude mice were randomly divided into Control (*n* ═ 4) and SAL (*n* ═ 4) groups. Following the method of Rong et al. [20], the SAL group was injected intraperitoneally with 0.3 mL of SAL solution (80 mg/kg), and the Control group received an equal dosage of PBS solution. The drug was administered once every two days. Subcutaneous tumors were measured using vernier calipers on days 7, 12, 17, 22, and 27. The mice were anesthetized and executed on the 27th day; tumors were excised, weighed, and photographed for recording.

### Immunohistochemistry

The OC tissues of nude mice were taken, treated with 4% paraformaldehyde, routinely dehydrated, and paraffin-embedded. Then, the wax blocks of the tissue specimens were routinely sliced (4–5 µm thickness), followed by Xylene dewaxing, gradient ethanol rehydration, and a microwave antigen repair. The sections were treated with Ki67 primary antibody (ab15580, 1:1000, Abcam) or Caspase-3 primary antibody (700182, 1:50, Invitrogen) and incubated at 37 ^∘^C for 1.5 h. The goat anti-rabbit IgG secondary antibody (31460, 1:10000, Invitrogen) was incubated for 20 min at 37 ^∘^C. DAB (Solarbio) was used to develop the color, and the color development was terminated with tap water. Mayer hematoxylin (Sigma-Aldrich) was used for re-staining, and neutral gum was used to seal the staining before observing it under a light microscope.

### Ethical statement

This study was approved by the Harbin Medical University Cancer Hospital Ethics Committee (2022-0906).

### Statistical analysis

Each experiment was performed a minimum of three times, and the data was recorded as the mean value with the corresponding standard deviation. SPSS 26.0 software (IBM SPSS Statistics 26) was used to process and statistically analyze the data. Student’s *t*-test was used to examine the differences between the two groups, and ANOVA was applied to make comparisons between sub-multiple groups. Prism software (Graphpad 9.0) was used for plotting. *P < 0.05 denotes that there is a significant difference.

## Results

### SAL suppresses OC cell proliferation and induces apoptosis

**Figure 1.**
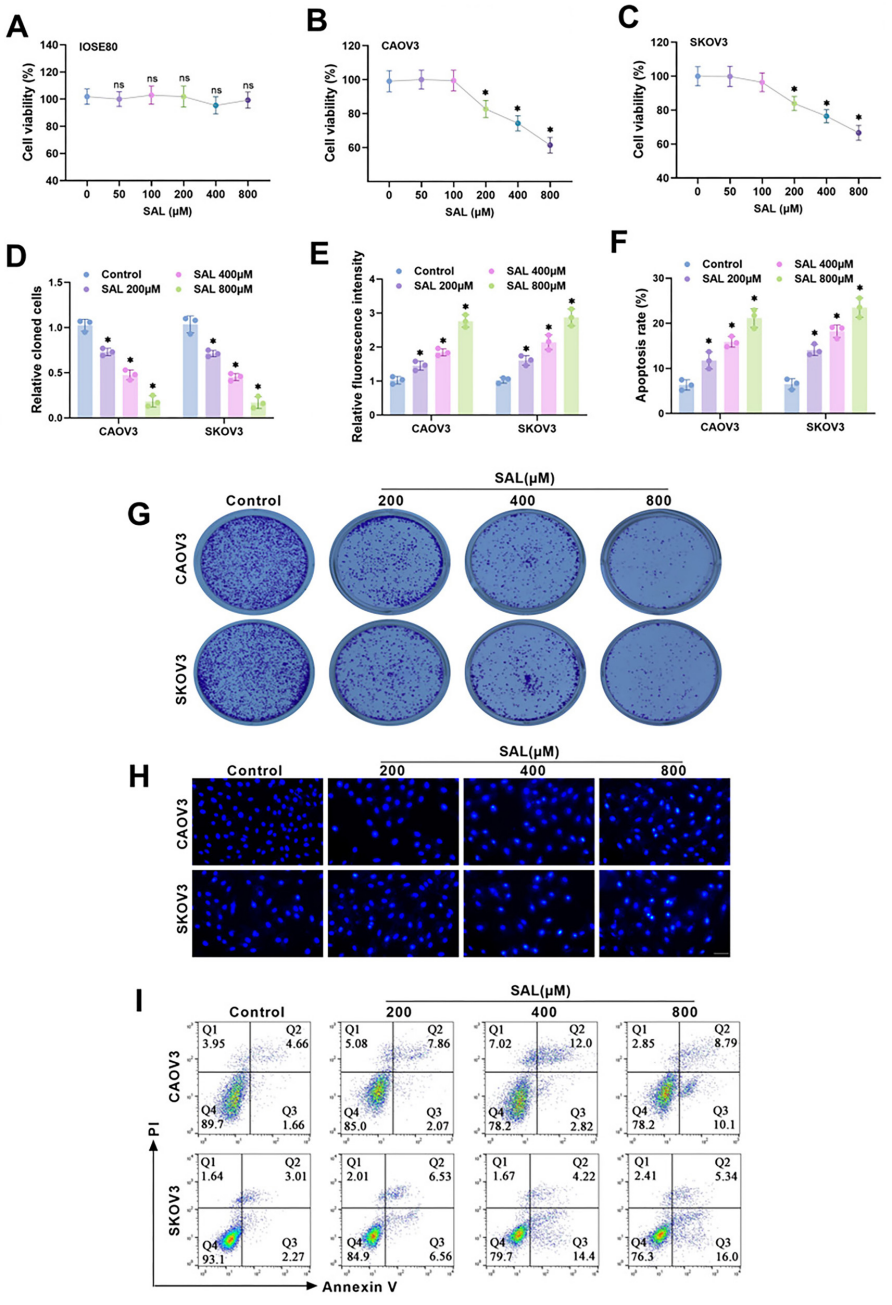
**SAL hinders OC cell proliferation and promotes apoptosis.** (A–C) The cell proliferation of IOSE80, CAOV3, and SKOV3 cells after treatment with SAL was monitored using the CCK-8 assay; (D and G) The colony formation rate of CAOV3 and SKOV3 cells was identified using the clone formation assay; (E and H) The apoptosis of CAOV3 and SKOV3 cells was detected using the Hoechst 33258 staining. Scale bar ═ 100 µm; (F and I) The apoptosis rate was examined utilizing flow cytometry. *n* ═ 3, *P < 0.05 vs Control. SAL: Salidroside; OC: Ovarian cancer; CCK-8: Cell counting kit-8.

The viability of cells was measured using the CCK-8 assay to assess the impact of SAL, and the results showed that SAL (200, 400, or 800 µM) treatment notably decreased the viability of CAOV3 and SKOV3 cells, but had no significant impact on IOSE80 cells (Figure 1A–1C). Therefore, we chose 200, 400, and 800-µM SAL for subsequent experiments. Clone formation experiments showed that SAL treatment significantly reduced the clone formation ability of CAOV3 and SKOV3 cells (Figure 1D and 1G). Not only that, we used Hoechst 33258 staining and flow cytometry to determine the influence of SAL on apoptosis. The data revealed a notable increase in fluorescence intensity (Figure 1E and 1H) and apoptosis rate (Figure 1F and 1I) among CAOV3 and SKOV3 cells following SAL treatment. These results suggested that SAL hindered the proliferation of OC cells and induced apoptosis in a dose-dependent manner.

### SAL inhibits OC cell migration, invasion, and EMT

**Figure 2.**
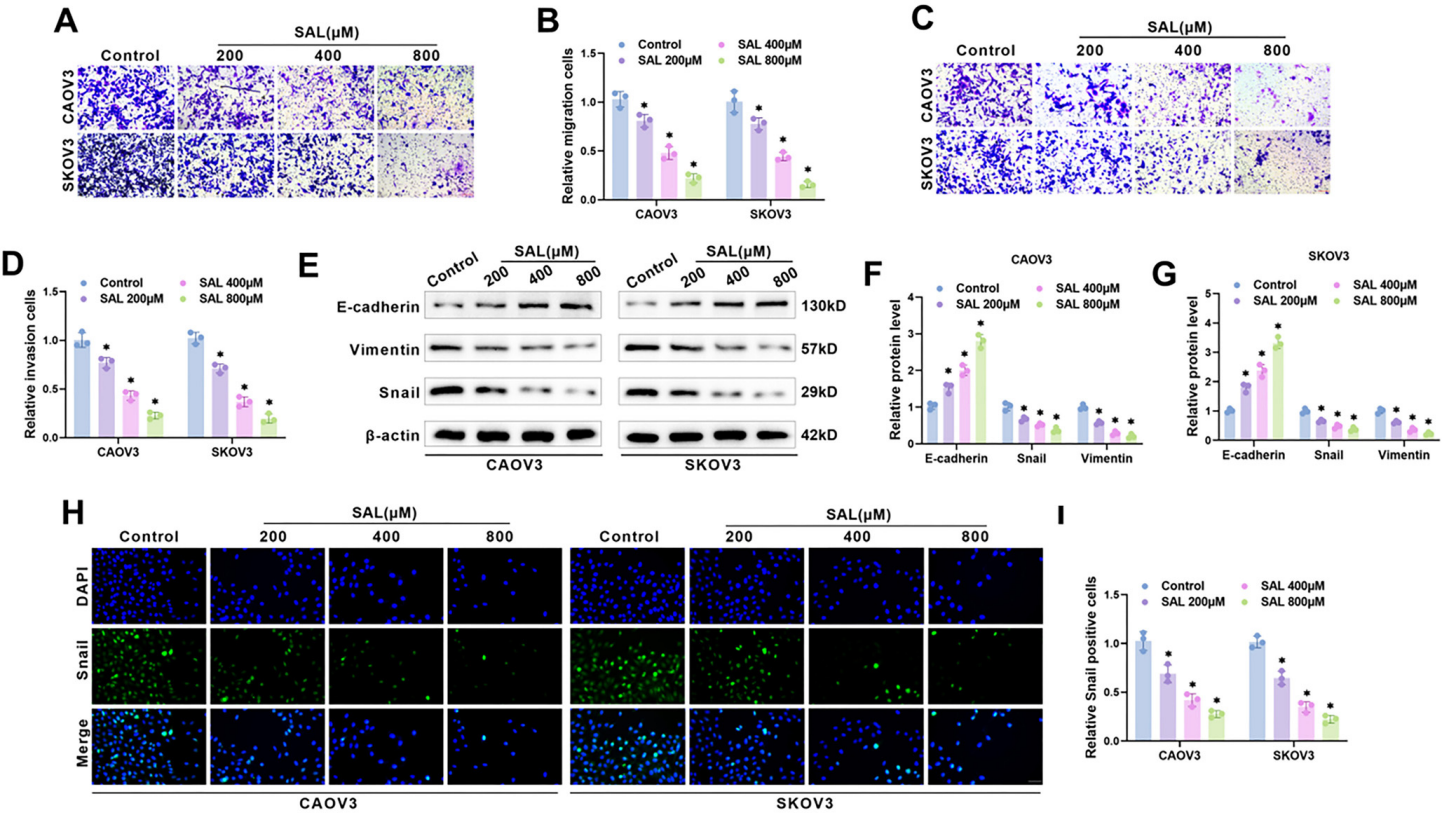
**SAL inhibits migration, invasion and EMT of OC cells.** (A–D) The amount of migration and invasion cells after treatment with SAL was calculated using the transwell assay. Scale bar ═ 100 µm; (E–G) Examining EMT-related protein levels in CAOV3 and SKOV3 cells through western blot; (H and I) Examining Snail expression in CAOV3 and SKOV3 cells through immunofluorescence. Scale bar ═ 100 µm. *n* ═ 3, *P < 0.05 vs Control. SAL: Salidroside; OC: Ovarian cancer; EMT: Epithelial–mesenchymal transition.

The Transwell assay was utilized to detect the impacts of SAL on cell migration and invasion capability, and we found that SAL markedly reduced the migration and invasion ability of CAOV3 and SKOV3 cells in a dose-dependent manner (Figure 2A–2D). Next, we used western blot to determine the levels of EMT-related proteins. Our findings revealed a notable increase in the level of E-cadherin protein in CAOV3 and SKOV3 cells after SAL treatment, while there is a notable decrease in the expression of both Snail and Vimentin (Figure 2E–2G). Not only that, we observed that SAL treatment led to a notable reduction of Snail-positive cells through immunofluorescence, consistent with the western blot results (Figure 2H and 2I). These results indicated that SAL was able to hinder the migration, invasion, and EMT of OC cells.

### SAL inhibits glycolysis in OC cells

**Figure 3.**
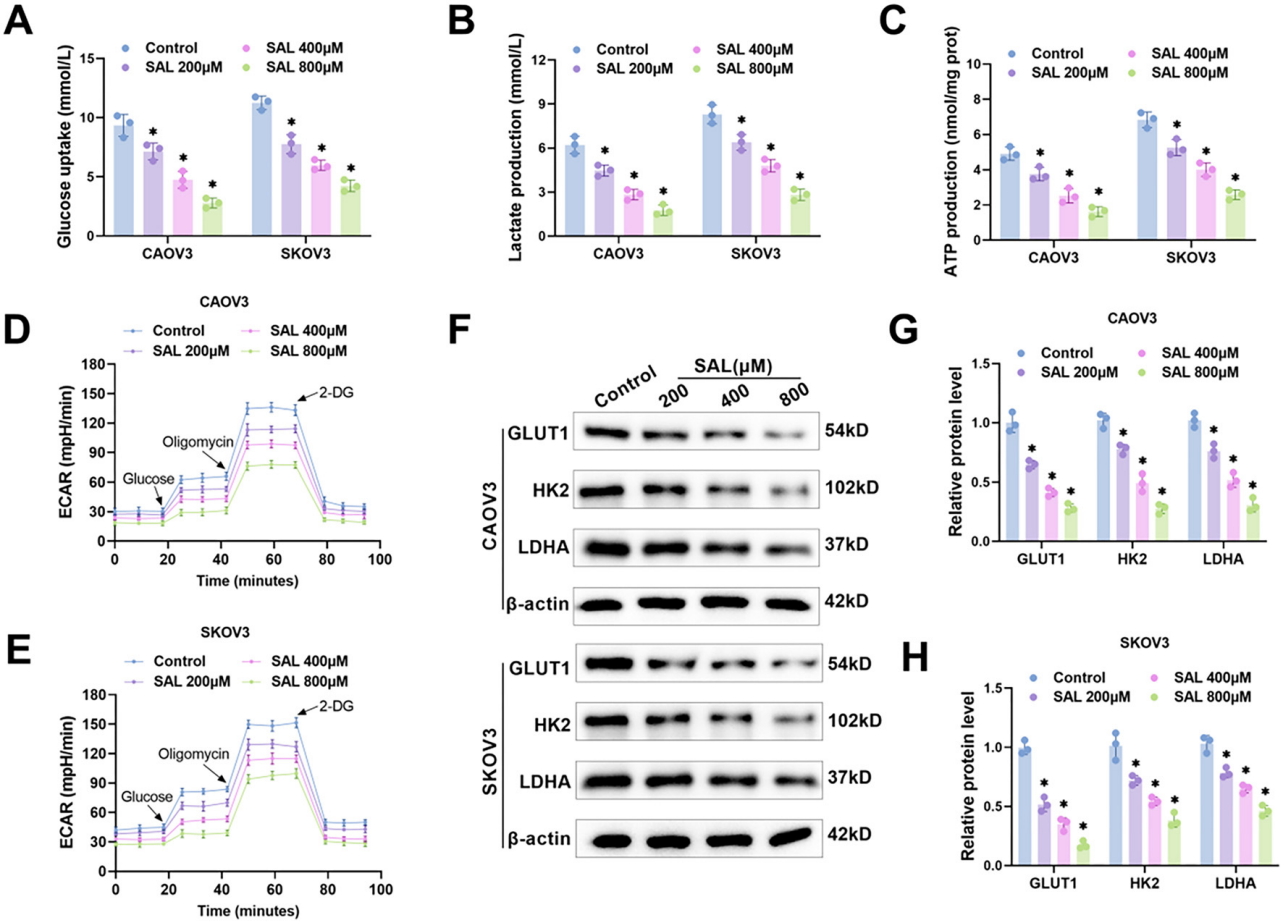
**SAL inhibits OC cell glycolysis.** (A–C) After treatment with SAL, the glucose consumption, lactate and ATP production in CAOV3 and SKOV3 cells were detected using different kits; (D and E) Examining the ECAR of OC cells using the XFe Seahorse glycolysis stress test kit; (F–H) Examining the levels of GLUT1, HK2, and LDHA protein through western blot. *n* ═ 3, *P < 0.05 vs Control. SAL: Salidroside; OC: Ovarian cancer; ECAR: Extracellular acidification rate; GLUT1: Glucose transporter type 1; HK2: Hexokinase 2; LDHA: Lactate dehydrogenase A.

In order to determine the effect of SAL on glycolysis in OC cells, we used different kits to detect changes in glucose consumption, lactate, and ATP production after SAL treatment. The results showed that SAL treatment notably decreased glucose consumption (Figure 3A) and lactate and ATP production (Figure 3B and 3C) in CAOV3 and SKOV3 cells, indicating that SAL inhibited OC cell glycolysis. Not only that, SAL treatment significantly reduced the ECAR of CAOV3 and SKOV3 cells (Figure 3D and 3E). In addition, the expression of key regulators of glycolysis, GLUT1, HK2, and LDHA, was markedly reduced in CAOV3 and SKOV3 cells after SAL treatment (Figure 3F–3H), further confirming that SAL inhibited OC cell glycolysis in a dose-dependent manner.

### SAL inhibits the STAT3/c-Myc pathway

**Figure 4.**
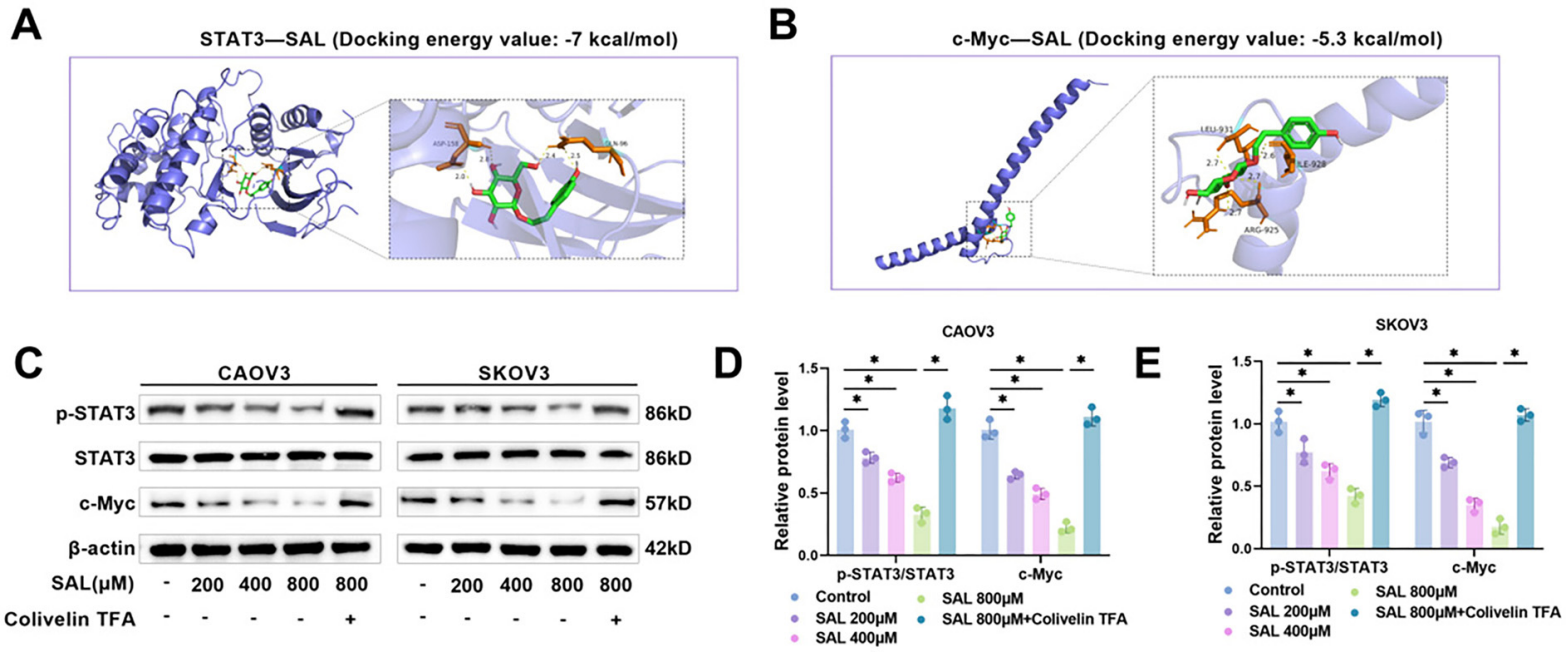
**SAL inhibits the STAT3/c-Myc pathway.** (A and B) The 3D structure of SAL and the STAT3 and c-Myc protein structures were obtained from the PubChem database and RCSB PDB database. Molecular docking of SAL with STAT3 and c-Myc proteins was verified using AutoDock software; (C–E) After treatment with SAL or Colivelin TFA, examining the levels of STAT3/c-Myc pathway-related proteins in CAOV3 and SKOV3 cells through western blot. *n* ═ 3, *P < 0.05. SAL: Salidroside; STAT3: Signal transducer and activator of transcription 3.

Studies have shown that STAT3/c-Myc is a key signaling pathway in the regulation of glycolysis [27]. To investigate whether SAL inhibits OC cell glycolysis through the STAT3/c-Myc pathway, we obtained the 3D structure of SAL from the PubChem database (https://pubchem.ncbi.nlm.nih.gov/). The STAT3 and c-Myc protein structures were obtained from the RCSB PDB database (https://www.rcsb.org/). The molecular docking of SAL with STAT3 and c-Myc proteins was verified using AutoDock software. It is generally accepted that docking energy values less than −4.25 kcal/mol indicate some binding activity between the two, less than −5.0 kcal/mol indicates good binding activity, and less than −7.0 kcal/mol indicates strong binding activity. Our results indicated that SAL had good binding activity with STAT3 and c-Myc with docking energy values of SAL and STATA3, SAL and c-Myc of −7 kcal/mol and −5.3 kcal/mol respectively, which indicated that SAL could directly target STAT3/c-Myc (Figure 4A and 4B). Next, we examined the levels of STAT3/c-Myc pathway-related proteins by western blot. According to the results, the levels of phosphorylated STAT3 and c-Myc expression were dose-dependently reduced in CAOV3 and SKOV3 cells after being treated with SAL, while the addition of the STAT3 agonist Colivelin TFA attenuated the effect of SAL, suggesting that SAL was able to inhibit the STAT3/c-Myc pathway (Figure 4C–4E).

### SAL inhibits OC cell glycolysis by inhibiting the STAT3/c-Myc pathway

**Figure 5.**
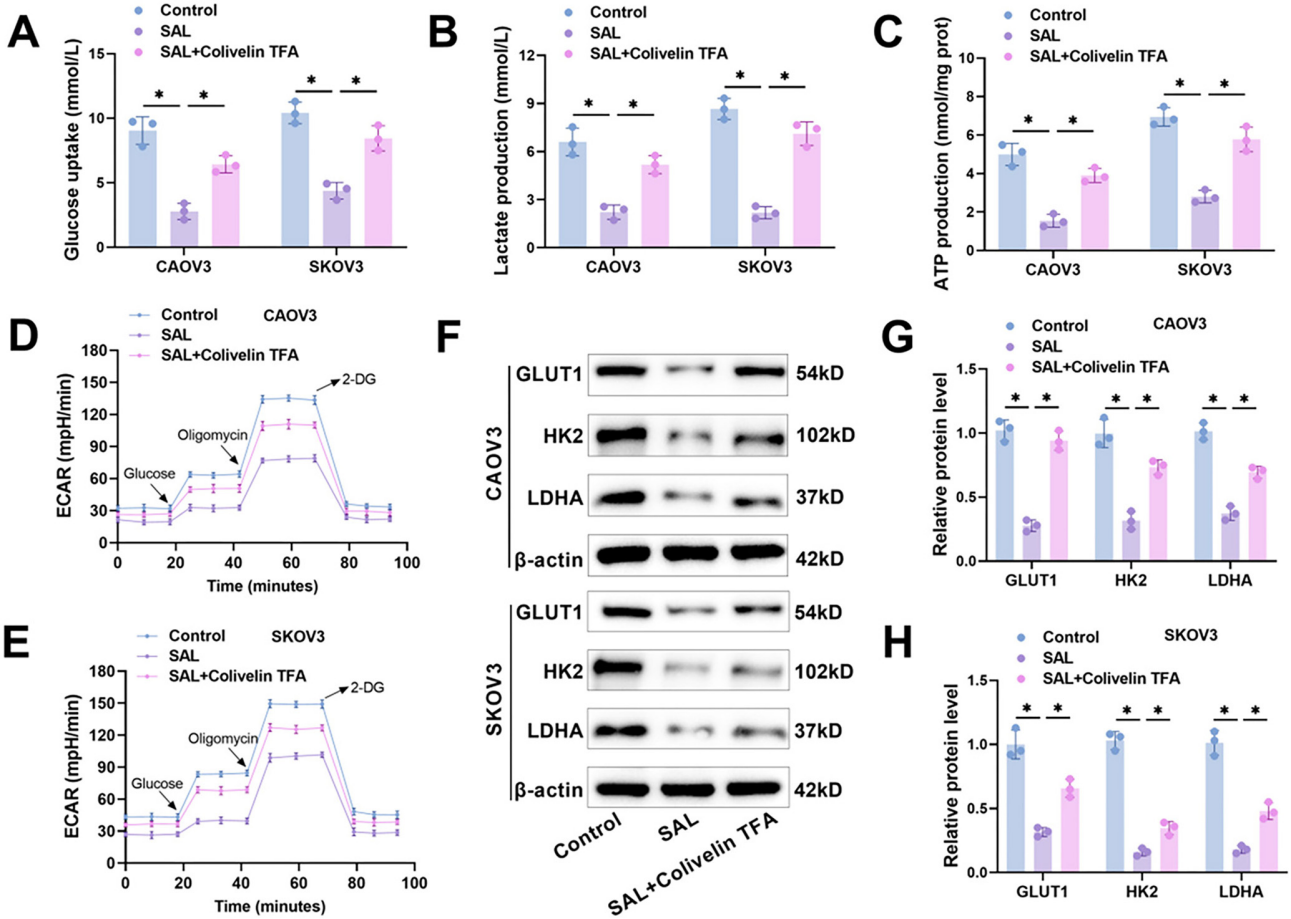
**SAL inhibits OC cell glycolysis by hindering the STAT3/c-Myc pathway.** (A–C) The glucose consumption, lactate and ATP production in CAOV3 and SKOV3 cells were detected using different kits; (D and E) Examining the ECAR of OC cells utilizing XFe Seahorse glycolysis stress test kit; (F–H) Examining the levels of GLUT1, HK2, and LDHA protein in CAOV3 and SKOV3 cells through western blot. *n* ═ 3, *P < 0.05. SAL: Salidroside; OC: Ovarian cancer; ECAR: Extracellular acidification rate; STAT3: Signal transducer and activator of transcription 3; GLUT1: Glucose transporter type 1; HK2: Hexokinase 2; LDHA: Lactate dehydrogenase A.

According to our previous experiments, 800 µM SAL was able to significantly inhibit OC cell glycolysis; therefore, we chose 800 µM SAL for the next study. Our research revealed that SAL resulted in reduced glucose consumption and hindered lactate and ATP production, which was attenuated by Colivelin TFA, suggesting that SAL may inhibit glycolysis through the STAT3/c-Myc pathway (Figure 5A–5C). SAL treatment resulted in a significant reduction of ECAR in CAOV3 and SKOV3 cells, which was partially restored by Colivelin TFA (Figure 5D and 5E). Not only that, SAL significantly decreased the expression of GLUT1, HK2, and LDHA in CAOV3 and SKOV3 cells, whereas Colivelin TFA reduced the effect of SAL, further confirming that SAL inhibits OC cell glycolysis by hindering the STAT3/c-Myc pathway (Figure 5F–5H).

### Activation of STAT3/c-Myc pathway attenuates proliferation inhibition and apoptosis promotion of OC cells by SAL

**Figure 6.**
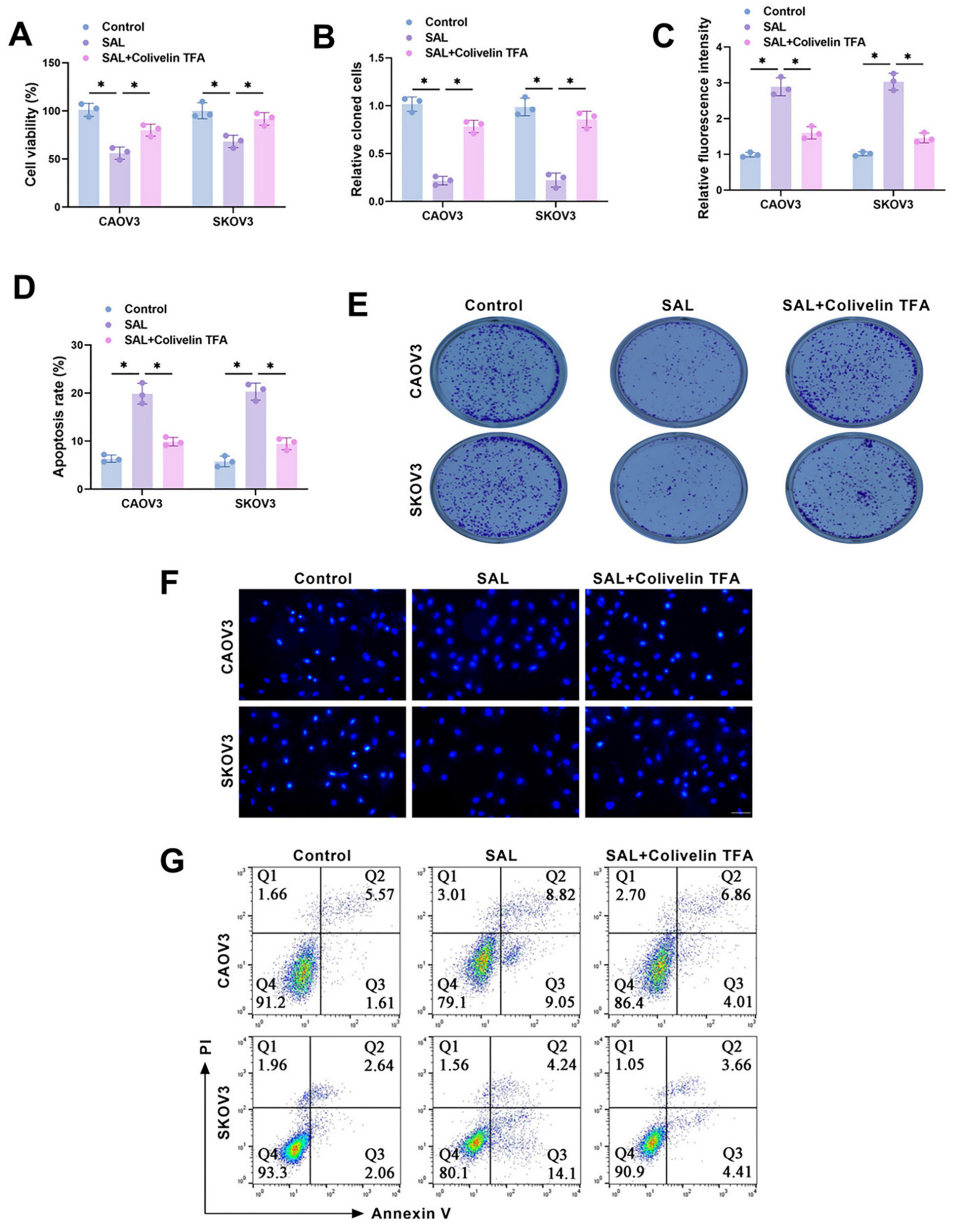
**Activation of STAT3/c-Myc pathway attenuates proliferation inhibition and apoptosis promotion of OC cells by SAL.** (A) Utilizing the CCK-8 assay monitored the cell proliferation of CAOV3 and SKOV3 cells after treatment with SAL or Colivelin TFA; (B and E) Colony formation rate was identified using the clone formation assay; (C and F) Apoptosis of CAOV3 and SKOV3 cells was determined utilizing the Hoechst 33258 staining. Scale bar ═ 100 µm; (D and G) The apoptosis rate was examined utilizing flow cytometry. *n* ═ 3, *P < 0.05. SAL: Salidroside; OC: Ovarian cancer; CCK-8: Cell counting kit-8; STAT3: Signal transducer and activator of transcription 3.

Next, we explored the impacts of activating the STAT3/c-Myc pathway on the malignant biological behaviors of OC cells. It was observed that SAL treatment notably decreased the viability of OC cells (Figure 6A) and inhibited the clone formation ability (Figure 6B and 6E), whereas Colivelin TFA impaired the effect of SAL. Hoechst 33258 staining showed that Colivelin TFA attenuated the promotive effect of SAL treatment on apoptosis (Figure 6C and 6F). Not only that, SAL treatment resulted in a notable rise in the apoptosis rate for CAOV3 and SKOV3 cells as observed through flow cytometry, while Colivelin TFA reduced this effect (Figures 6D and 6G). The above results revealed that the activation of the STAT3/c-Myc pathway lessens the impact of SAL on inhibiting OC cell proliferation and promoting apoptosis.

### STAT3/c-Myc pathway activation attenuates the inhibition of SAL on the migration, invasion, and EMT of OC cells

**Figure 7.**
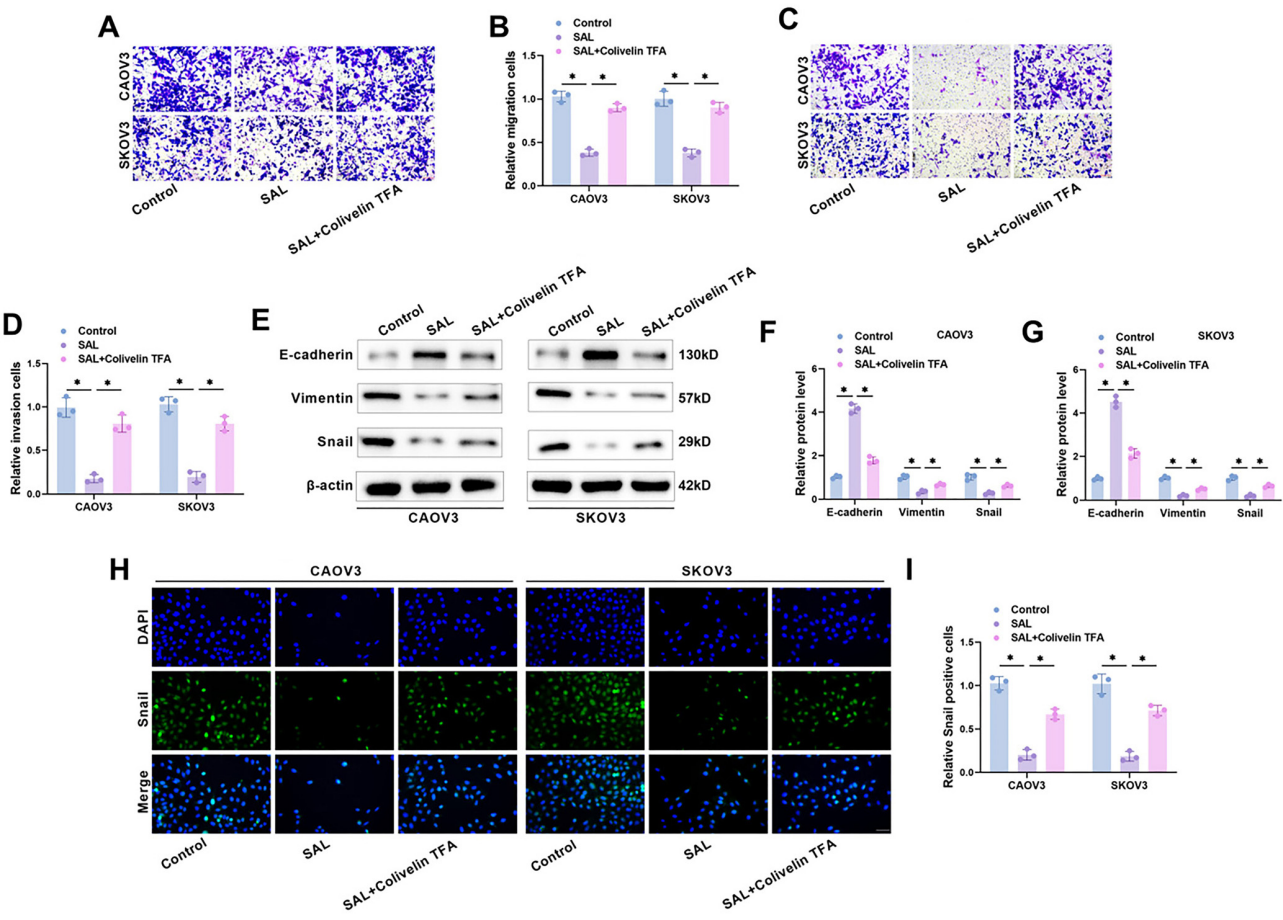
**STAT3/c-Myc pathway activation attenuates the inhibitory effect of SAL on the migration, invasion and EMT of OC cells.** (A–D) The amount of migration and invasion cells after treatment with SAL or Colivelin TFA was calculated using the transwell assay. Scale bar ═ 100 µm; (E–G) Examining EMT-related protein levels in CAOV3 and SKOV3 cells through western blot; (H and I) Examining Snail expression in CAOV3 and SKOV3 cells through immunofluorescence. Scale bar ═ 100 µm. *n* ═ 3, *P < 0.05. SAL: Salidroside; OC: Ovarian cancer; EMT: Epithelial–mesenchymal transition; STAT3: Signal transducer and activator of transcription 3.

SAL treatment notably decreased the migration and invasion capacities of CAOV3 and SKOV3 cells, while the inhibition of SAL treatment on cell migration and invasion was attenuated with the addition of Colivelin TFA, as indicated by Transwell assay results (Figure 7A–7D). Western blot results revealed that after SAL treatment, the expression level of E-cadherin in CAOV3 and SKOV3 cells was significantly increased, while the expression levels of Snail and Vimentin were markedly decreased, and the effect of SAL was weakened by Colivelin TFA (Figure 7E–7G). Not only that, immunofluorescence results indicated that SAL reduced the number of Snail-positive cells, and the addition of Colivelin TFA was able to attenuate this phenomenon (Figure 7H and 7I), suggesting that SAL may inhibit OC cell migration, invasion, and EMT through the STAT3/c-Myc pathway.

### SAL inhibits tumor growth and glycolysis in vivo

**Figure 8.**
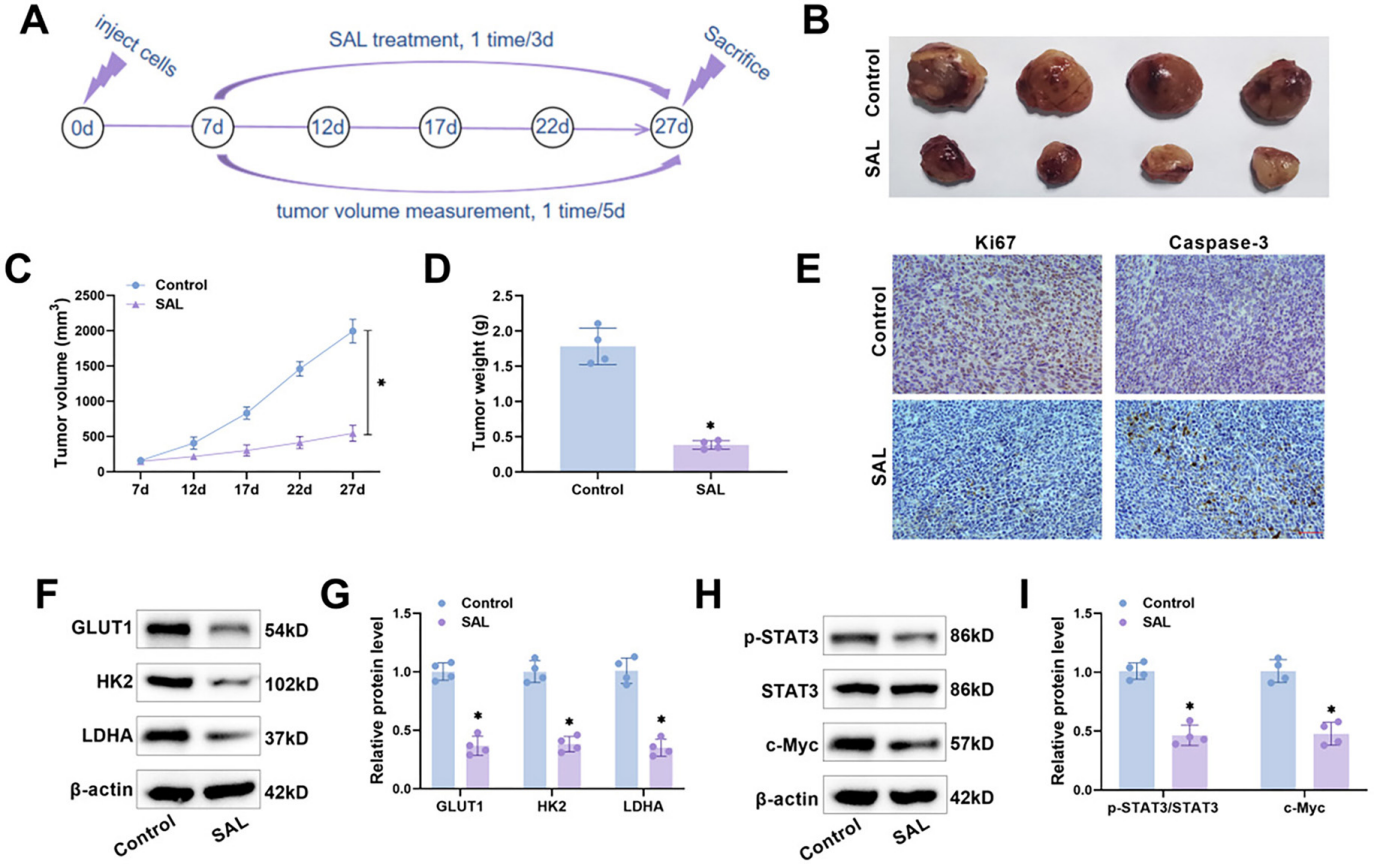
**SAL hinders tumor growth and glycolysis in vivo.** (A) Flowchart of the construction of the subcutaneous transplantation tumor model in nude mice. Nude mice were executed after anesthesia on day 27; tumor tissues were excised, photographed (B) and weighed (D); (C) Subcutaneous tumor dimensions were assessed on days 7, 12, 17, 22, and 27 using a vernier caliper in order to determine the tumor volume; (E) Analyzing Ki-67 and Caspase-3 expression in tumor tissues via immunohistochemistry. Scale bar ═ 50 µm; (F and G) Examining the levels of GLUT1, HK2, and LDHA protein in tumor tissues through western blot. (H and I) Examining STAT3/c-Myc pathway-related protein expression in tumor tissues through western blot. *n* ═ 4, *P < 0.05 vs Control. SAL: Salidroside; STAT3: Signal transducer and activator of transcription 3; GLUT1: Glucose transporter type 1; HK2: Hexokinase 2; LDHA: Lactate dehydrogenase A.

Finally, a xenograft tumor model was constructed according to the process shown in Figure 8A to explore the influence of SAL on tumor growth in nude mice. The results indicated that the volume and weight of the tumors in vivo were significantly reduced after the injection of SAL (Figure 8B–8D), revealing that SAL could effectively inhibit tumor growth. We used immunohistochemistry to determine the expression of Ki67 and Caspase-3 in OC tissues, and the findings revealed a notable decrease in Ki67 expression and an increase in Caspase-3 expression following SAL injection (Figure 8E). In addition, SAL treatment led to a marked decrease in the levels of GLUT1, HK2, and LDHA proteins in OC tissues (Figure 8F and 8G). Notably, SAL resulted in a marked reduction in STAT3 phosphorylation levels and c-Myc expression in OC tissues (Figure 8H and 8I), further suggesting that SAL inhibits OC malignant progression and glycolysis by regulating the STAT3/c-Myc pathway.

**Figure 9.**
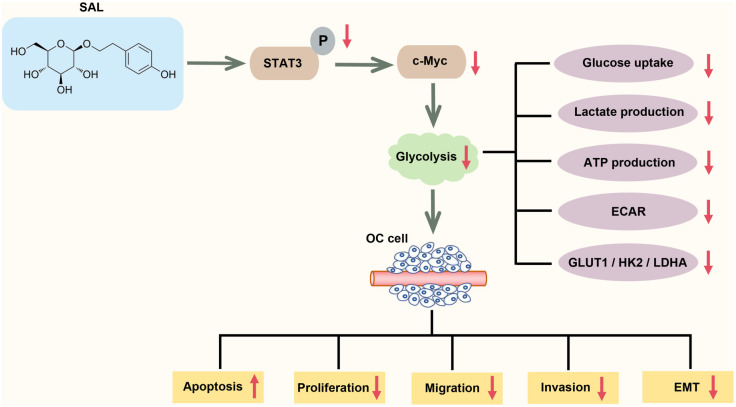
**Salidroside interferes glycolytic processes by inhibiting the STAT3/c-Myc pathway leading to the inhibition of malignant biological behaviors and progresses of OC cells.** OC: Ovarian cancer; STAT3: Signal transducer and activator of transcription 3.

## Discussion

SAL, a crucial ingredient found in *Rhodiola rosea,* has a wide range of pharmacological properties, such as anti-inflammatory, antioxidant, and anti-tumor activities, and has been a hotspot in the research of new natural anticancer drugs in recent years. It has been shown that SAL dose-dependently reduces colorectal cancer cell viability and promotes apoptosis and autophagy through the inhibition of the PI3K/Akt/mTOR pathway [28]. Qi et al. [29] showed that SAL hindered the proliferation and migration of gastric cancer cells by down-regulating the ROS-mediated Src-related signaling pathway. In this research, we found that SAL notably reduced the viability of OC cells, impeded cell migration and invasion, and induced apoptosis, with the effects being dependent on the dosage, a result consistent with that reported by Yu et al. [22].

EMT is a biological process in which cobblestone-like epithelial phenotype cells are transformed into spindle-shaped mesenchymal phenotype cells [30]. Previous studies have shown that EMT is implicated in tumorigenesis and metastasis in addition to embryogenesis, organ development, and tissue formation [31]. The EMT process enhances the migration and invasion of tumor cells while also allowing them to develop problems such as immunosuppression and drug resistance [32, 33]. Therefore, the inhibition of EMT is an important direction for the treatment of highly invasive and metastatic types of cancer. After the occurrence of EMT in cancer cells, E-cadherin expression is downregulated, while proteins, such as mesenchymal markers N-cadherin, Vimentin, and Snail are upregulated [34]. Our results indicated that E-cadherin protein expression was upregulated in OC cells after SAL treatment, while both Snail and Vimentin protein expressions were downregulated, suggesting that SAL inhibited the EMT process in OC cells.

Glycolysis is known to produce ATP necessary for the continued proliferation and metastasis of cancer cells and is one of the most important ways for tumor cells to obtain energy [35, 36]. Aerobic glycolysis, also known as the Warburg effect, causes tumor cells to convert glucose into lactic acid even when oxygen levels are adequate. This process is characterized by a notable surge in glucose uptake and lactate production [37, 38]. The expression of glycolytic enzymes, including LDHA, HK2, phosphoglycerol kinase 1 (PGK1), and pyruvate kinase isoform M2 (PKM2), as well as glucose and lactate transport proteins, such as GLUT1 and monocarboxylic acid transporter 4 (MCT4), are upregulated to enhance glucose uptake during glycolysis in cancer cells [39, 40]. In this research, we found that SAL inhibited the glycolysis process in OC cells, resulting in a significant decrease in glucose consumption, lactate and ATP production, and ECAR, suggesting that SAL may exert an inhibitory effect on cancer by inhibiting glycolysis. Dai et al. [41] similarly found that SAL inhibited proliferation, induced apoptosis, and decreased the activities of glycolytic enzymes, such as PKM2 and enolase 1 (ENO1) and the expression of GLUT1 in gastric cancer cells.

STAT3 plays a crucial role as a transcription factor in controlling key biological processes like cell growth, differentiation, programmed cell death, and glycolysis [42]. c-Myc is one of the downstream genes of STAT3 and is a crucial factor in energy metabolism and glycolysis [43]. Zhao et al. [44] found that Alpinetin inhibited STAT3 signaling, which in turn inhibited OC cell proliferation and migration. Zhang et al. [45] showed that Fucoxanthin hinders the malignant biological behavior and glycolysis of OC cells by hindering the STAT3/c-Myc pathway. Therefore, we hypothesized that SAL may be involved in inhibiting the malignant progression of OC by modulating the STAT3/c-Myc pathway. We found that the level of STAT3 phosphorylation and c-Myc expression were dose-dependently reduced in OC cells after SAL treatment, whereas STAT3 agonist intervention attenuated the effect of SAL, suggesting that SAL inhibited the STAT3/c-Myc pathway. Additionally, STAT3 agonists impaired the inhibition of SAL on the malignant biological progression and glycolysis process of OC cells, and reduced the apoptotic rate. Notably, SAL similarly reduced the expression level of STAT3/c-Myc pathway-related proteins in mice and effectively inhibited OC tumor growth and glycolysis processes.

## Conclusion

SAL reduced OC cell viability, migration, and invasiveness, induced apoptosis, and inhibited cellular EMT by inhibiting glycolytic processes through the STAT3/c-Myc pathway (Figure 9). Notably, SAL effectively inhibited the growth of OC tumors in mice. The present study elucidated the potential mechanism by which SAL exerts anti-tumor effects in OC; however, it is necessary to conduct additional evaluations to ensure the safety and efficacy of SAL in clinical applications. In conclusion, the findings of this study suggest that SAL has the potential to be used as a novel approach for the clinical management of OC.

## Data Availability

The data that support the findings of this study are available from the corresponding author, [XLF], upon reasonable request.

## References

[1] Sambasivan S (2022). Epithelial ovarian cancer: review article. Cancer Treat Res Commun.

[2] Stewart C, Ralyea C, Lockwood S (2019). Ovarian cancer: an integrated review. Semin Oncol Nurs.

[3] Zhang R, Siu MKY, Ngan HYS, Chan KKL (2022). Molecular biomarkers for the early detection of ovarian cancer. Int J Mol Sci.

[4] Lheureux S, Braunstein M, Oza AM (2019). Epithelial ovarian cancer: Evolution of management in the era of precision medicine. CA Cancer J Clin.

[5] Kossaï M, Leary A, Scoazec JY, Genestie C (2018). Ovarian cancer: a heterogeneous disease. Pathobiology.

[6] Armstrong DK, Alvarez RD, Bakkum-Gamez JN, Barroilhet L, Behbakht K, Berchuck A (2021). Ovarian Cancer, Version 2. 2020, NCCN clinical practice guidelines in oncology. J Natl Compr Canc Netw.

[7] Moufarrij S, Dandapani M, Arthofer E, Gomez S, Srivastava A, Lopez-Acevedo M (2019). Epigenetic therapy for ovarian cancer: promise and progress. Clin Epigenet.

[8] Yang L, Xie HJ, Li YY, Wang X, Liu XX, Mai J (2022). Molecular mechanisms of platinum-based chemotherapy resistance in ovarian cancer (Review). Oncol Rep.

[9] Eisenhauer EA (2017). Real-world evidence in the treatment of ovarian cancer. Ann Oncol.

[10] Abbott M, Ustoyev Y (2019). Cancer and the Immune System: the history and background of immunotherapy. Semin Oncol Nurs.

[11] Zhang Y, Zhang Z (2020). The history and advances in cancer immunotherapy: understanding the characteristics of tumor-infiltrating immune cells and their therapeutic implications. Cell Mol Immunol.

[12] Yang Z, Zhang Q, Yu L, Zhu J, Cao Y, Gao X (2021). The signaling pathways and targets of traditional Chinese medicine and natural medicine in triple-negative breast cancer. J Ethnopharmacol.

[13] He Q, Liu C, Wang X, Rong K, Zhu M, Duan L (2023). Exploring the mechanism of curcumin in the treatment of colon cancer based on network pharmacology and molecular docking. Front Pharmacol.

[14] Lu Y, Deng B, Xu L, Liu H, Song Y, Lin F (2022). Effects of Rhodiola Rosea supplementation on exercise and sport: a systematic review. Front Nutr.

[15] Nabavi SF, Braidy N, Orhan IE, Badiee A, Daglia M, Nabavi SM (2016). Rhodiola rosea L. Alzheimer’s disease: from farm to pharmacy. Phytoth Res.

[16] Qu B, Liu X, Liang Y, Zheng K, Zhang C, Lu L (2022). Salidroside in the treatment of NAFLD/NASH. Chem Biodivers.

[17] Magani SKJ, Mupparthi SD, Gollapalli BP, Shukla D, Tiwari AK, Gorantala J (2020). Salidroside—can it be a multifunctional drug?. Curr Drug Metab.

[18] Liu RH, Ma TF, Yang Q, Xiao WC, Yin L, Yin M (2023). Salidroside suppresses proliferation and migration in prostate cancer via the PI3K/AKT pathway. Cancer Biomark.

[19] Wen Z, Liu T, Zhang Y, Yue Q, Meng H, He Y (2023). Salidroside regulates tumor microenvironment of non-small cell lung cancer via Hsp70/Stub1/Foxp3 pathway in Tregs. BMC Cancer.

[20] Rong L, Li Z, Leng X, Li H, Ma Y, Chen Y (2020). Salidroside induces apoptosis and protective autophagy in human gastric cancer AGS cells through the PI3K/Akt/mTOR pathway. Biomed Pharmacother.

[21] Yuetong L, Shangzhu L, Qinglin H, Pingping H (2020). Salidroside inhibits proliferation, migration and invasion of human pancreatic cancer PANC1 and SW1990 cells through the AKT and ERK signaling pathway. Pharmazie.

[22] Yu G, Li N, Zhao Y, Wang W, Feng XL (2018). Salidroside induces apoptosis in human ovarian cancer SKOV3 and A2780 cells through the p53 signaling pathway. Oncol Lett.

[23] Hillmer EJ, Zhang H, Li HS, Watowich SS (2016). STAT3 signaling in immunity. Cytokine Growth Factor Rev.

[24] Zou S, Tong Q, Liu B, Huang W, Tian Y, Fu X (2020). Targeting STAT3 in cancer immunotherapy. Mol Cancer.

[25] Sipos F, Firneisz G, Műzes G (2016). Therapeutic aspects of c-MYC signaling in inflammatory and cancerous colonic diseases. World J Gastroenterol.

[26] Ala M (2022). Target c-Myc to treat pancreatic cancer. Cancer Biol Ther.

[27] Zhang S, Li J, Xie P, Zang T, Shen H, Cao G (2019). STAT3/c-Myc Axis-mediated metabolism alternations of inflammation-related glycolysis involve with colorectal carcinogenesis. Rejuvenation Res.

[28] Fan XJ, Wang Y, Wang L, Zhu M (2016). Salidroside induces apoptosis and autophagy in human colorectal cancer cells through inhibition of PI3K/Akt/mTOR pathway. Oncol Rep.

[29] Qi Z, Tang T, Sheng L, Ma Y, Liu Y, Yan L (2018). Salidroside inhibits the proliferation and migration of gastric cancer cells via suppression of SRC-associated signaling pathway activation and heat shock protein 70 expression. Mol Med Rep.

[30] Manfioletti G, Fedele M (2023). Epithelial-mesenchymal transition (EMT). Int J Mol Sci.

[31] Mittal V (2018). Epithelial mesenchymal transition in tumor metastasis. Annu Rev Pathol.

[32] Dongre A, Weinberg RA (2019). New insights into the mechanisms of epithelial-mesenchymal transition and implications for cancer. Nat Rev Mol Cell Biol.

[33] Zhang Y, Weinberg RA (2018). Epithelial-to-mesenchymal transition in cancer: complexity and opportunities. Front Med.

[34] Serrano-Gomez SJ, Maziveyi M, Alahari SK (2016). Regulation of epithelial-mesenchymal transition through epigenetic and post-translational modifications. Mol Cancer.

[35] Paul S, Ghosh S, Kumar S (2022). Tumor glycolysis, an essential sweet tooth of tumor cells. Semin Cancer Biol.

[36] Abbaszadeh Z, Çeşmeli S, Biray Avcı Ç (2020). Crucial players in glycolysis: cancer progress. Gene.

[37] Liberti MV, Locasale JW (2016). The Warburg effect: how does it benefit cancer cells?. Trends Biochem Sci.

[38] Kocianova E, Piatrikova V, Golias T (2022). Revisiting the Warburg effect with focus on lactate. Cancers (Basel).

[39] Vaupel P, Multhoff G (2021). Revisiting the Warburg effect: historical Dogma versus current understanding. J Physiol.

[40] Fukushi A, Kim HD, Chang YC, Kim CH (2022). Revisited metabolic control and reprogramming cancers by means of the Warburg effect in tumor cells. Int J Mol Sci.

[41] Dai Z, Zhang X, Li W, Tang J, Pan T, Ma C (2021). Salidroside induces apoptosis in human gastric cancer cells via the downregulation of ENO1/PKM2/GLUT1 expression. Biol Pharm Bull.

[42] Yu H, Lee H, Herrmann A, Buettner R, Jove R (2014). Revisiting STAT3 signalling in cancer: new and unexpected biological functions. Nat Rev Cancer.

[43] Khan F, Pandey P, Verma M, Upadhyay TK (2024). Terpenoid-mediated targeting of STAT3 signaling in cancer: an overview of preclinical studies. Biomolecules.

[44] Zhao X, Guo X, Shen J, Hua D (2018). Alpinetin inhibits proliferation and migration of ovarian cancer cells via suppression of STAT3 signaling. Mol Med Rep.

[45] Zhang Z, Wang Y, Li J (2023). Fucoxanthin suppresses the malignant progression of ovarian cancer by inactivating STAT3/c-Myc signaling. Am J Transl Res [Internet].

